# Biochemistry and toxicology of proteins and peptides puriﬁed from the venom of *Vipera berus berus*

**DOI:** 10.1016/j.toxcx.2022.100131

**Published:** 2022-06-12

**Authors:** Jüri Siigur, Ene Siigur

**Affiliations:** Laboratory of Environmental Toxicology, National Institute of Chemical Physics and Biophysics, Akadeemia Tee 23, 12618, Tallinn, Estonia

**Keywords:** Vipera berus barani, Vipera berus berus (Common viper), Vipera berus bosniensis, Vipera berus sachalinensis, Vipera berus marasso, Snake venom, ACE, angiotensin converting enzyme, AspP, aspartic protease, BAEE, benzoylarginine ethyl ester, BNP, B-type natriuretic peptide, BPP, bradykinin potentiating peptides, CNP, C-type natriuretic peptide, CRISP, cysteine rich secretory protein, CTL, C-type lectin/snaclec, Hyal, hyaluronidase, KUN, Kunitz type proteinase inhibitor, LAAO, L-amino acid oxidase, MALDI-TOF MS, matrix-assisted laser desorption ionization/time of flight mass spectrometry, 5′-NT, 5′- nucleotidase, NGF, nerve growth factor, NP, natriuretic peptide, PDE, phosphodiesterase, PLA_2_, phospholipase A_2_, PLB, phospholipase B, Pro-Phe-Arg-MCA, Pro-Phe-Arg-4-methylcoumarinyl-7-amide, QC, glutaminyl-peptide cyclotransferase, SVMP, snake venom metalloproteinase, SVSP, snake venom serine proteinase, TAME, tosylarginine methyl ester, VBFXAE, *V. berus* factor X activating enzyme, VEGF, vascular endothelial growth factor

## Abstract

The isolation and characterization of individual snake venom components is important for a deeper understanding of the pathophysiology of envenomation and for improving the therapeutic procedures of patients. It also opens possibilities for the discovery of novel toxins that might be useful as tools for understanding cellular and molecular processes. The variable venom composition, toxicological and immunological properties of the common vipers (*Vipera berus berus*) have been reviewed. The combination of venom gland transcriptomics, bottom-up and top-down proteomics enabled comparison of common viper venom proteomes from multiple individuals. *V. b. berus* venom contains proteins and peptides belonging to 10–15 toxin families: snake venom metalloproteinase, phospholipases A_2_ (PLA_2_), snake venom serine proteinase, aspartic protease, L-amino acid oxidase (LAAO), hyaluronidase, 5′-nucleotidase, glutaminyl-peptide cyclotransferase, disintegrin, C-type lectin (snaclec), nerve growth factor, Kunitz type serine protease inhibitor, snake venom vascular endothelial growth factor, cysteine-rich secretory protein, bradykinin potentiating peptide, natriuretic peptides. PLA_2_ and LAAO from *V. b. berus* venom produce more pronounced cytotoxic effects in cancer cells than normal cells, via induction of apoptosis, cell cycle arrest and suppression of proliferation. Proteomic data of *V. b. berus* venoms from different parts of Russia and Slovakian Republic have been compared with analogous data for *Vipera nikolskii* venom. Proteomic studies demonstrated quantitative differences in the composition of *V. b. berus* venom from different geographical regions. Differences in the venom composition of *V. berus* were mainly driven by the age, sex, habitat and diet of the snakes. The venom variability of *V. berus* results in a loss of antivenom efficacy against snakebites. The effectiveness of antibodies is discussed. This review presents an overview with a special focus on different toxins that have been isolated and characterized from the venoms of *V. b. berus.* Their main biochemical properties and toxic actions are described.

## Highlights

• *Vipera berus berus* venom composition is variable among different populations.
• Venom contains about 15 protein/peptide families.
• It disturbs blood coagulation inducing pro- or anticoagulant effects.
• Venom contains different types of blood factor X activators.
• PLA_2_ and L-*amino* acid oxidase produce cytotoxic effects in cancer cells.

## Graphical abstract

**Figure:**
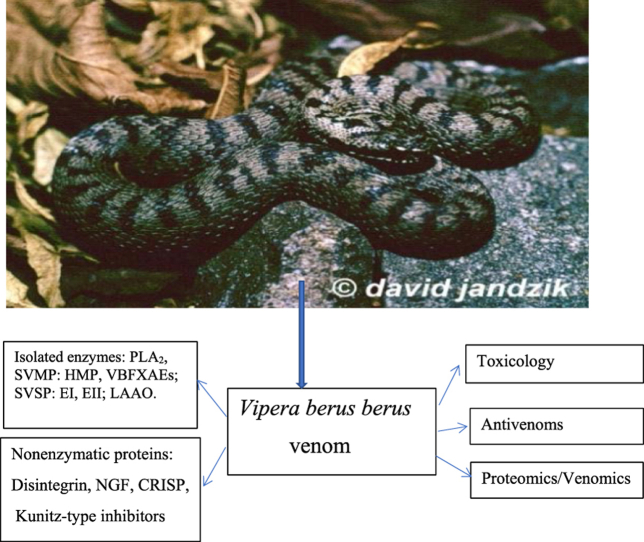

## 1 Introduction

Family Viperidae (Vipers, 374 species) is divided into three subfamilies: Azemiopinae (2 species), Viperinae (True Vipers, 101 species), and Crotalinae (Pit Vipers, 271 species) (data taken from www.reptile-database.org). A bibliographic search to the keyword “*Vipera berus”* in PubMed identified 201 hits between the years 1909 and 2021. The European adder (*Vipera berus*) is a small, stout-bodied snake that has a huge distribution area covering large parts of Europe and Asia. *Vipera berus* is arranged in five subspecies: *Vipera berus barani* (Böhme and Joger, 1983), *Vipera berus berus* (LINNAEUS 1758), *Vipera berus bosniensis* (BOETTGER 1889), *Vipera berus marasso* (POLLINI 1818), *Vipera berus sachalinensis* (TZAREVSKY 1917) (www.reptile-database.org).

*Vipera berus* is the most widely distributed terrestrial snake on the planet that occupies Eastern Europe, Western Europe, Central Europe, Central Asia, and East Asia.

**Vipera berus berus** snakes are found on: Norway, Sweden, Finland, France, Denmark, Germany, Austria, Switzerland, N Italy, Belgium, Netherlands, England (UK), Poland, Czech Republic (formerly Czechoslovakia), Hungary, Romania, Belarus, Bulgaria, Albania, Slovakia, Croatia, Slovenia, Macedonia, Bosnia-Hercegovina, Monte Negro, Serbia, Estonia, Latvia, Lithuania, Russia, Ukraine, Mongolia, Kazakhstan, North Korea, NW China (N Xinjiang, Jilin).

### 1.1 Vipera berus barani

NW Turkey; Type locality ca. 60 km N Adapazari, Turkey.

### 1.2 Vipera berus bosniensis

Bosnia, Croatia, Serbia, Macedonia, Montenegro, N Albania, N Greece, Hungary; *V. berus bosniensis* occupies two distinct habitat types: the lowlands of southwestern Hungary, northern Croatia, northern Serbia, and the mountains in the interior of the Balkan Peninsula south to Greece.

### 1.3 Vipera berus marasso

S Germany, Austria, N Italy. Type locality: Legnago, Prov. Verona, Po region (Contorni di Legnago).

### 1.4 Vipera berus sachalinensis

NE China, North Korea, Russia (Amur Oblast, Primorskye Kray and Khabarovsk Kray, Sakhalin = Sakhalin Island) (www.reptile-database.org).

**Fig. 1:**
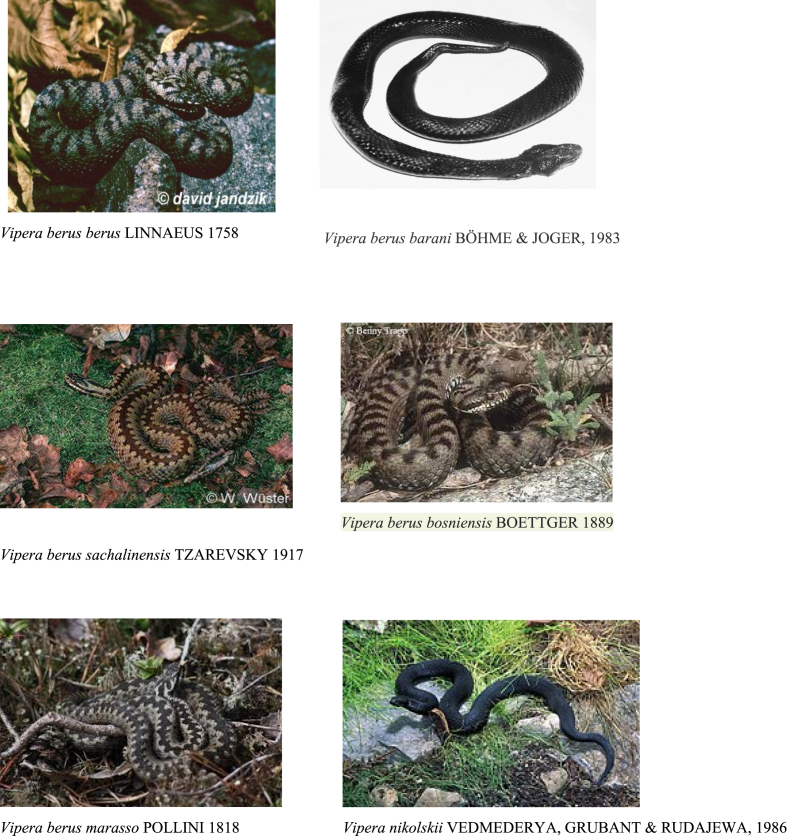
Photos of *Vipera berus* subspecies and *Vipera nikolskii* snakes.

**Fig. 2:**
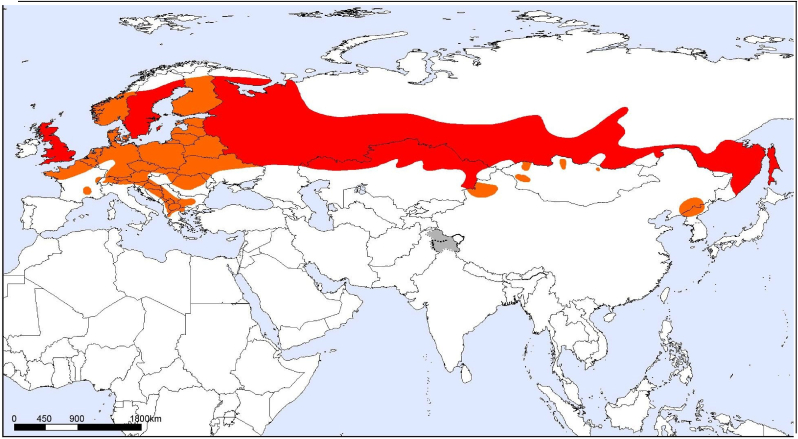
The distribution map of *Vipera berus* snakes. The definitions of Category 1 and 2 risk classes are contained in the WHO Guidelines on the Production, Control and Regulation of Snake Antivenom lmmunoglobulins. Areas shaded in red on maps indicate that a species is considered to be of Category 1importance in that part of its range, whereas areas shaded in orange indicate Category 2 importance. (For interpretation of the references to colour in this figure legend, the reader is referred to the Web version of this article.)

The photos of *Vipera berus* subspecies are presented on Fig. 1. “*Vipera berus”* as the name of the snake has been used in many papers without mentioning the subspecies. The distribution map of *Vipera berus* snakes is shown on Fig. 2.

Ursenbacher et al. (2006) examined the phylogeography of a widespread snake species (*Vipera berus*) inhabiting Europe up to the Arctic Circle. The analysis of the mitochondrial DNA sequence variation in 1043 bp of the cytochrome *b* gene and in 918 bp of the noncoding control region was performed with phylogenetic approaches. Phylogenetic analysis showed that *V. berus* is divided into three major clades: an Italian, a Balkan and a Northern (from France to Russia) clades. Cui et al. (2016) described the morphological characteristics and molecular phylogeny of *V. berus* snakes from two areas of Southern Altay Mountains. A phylogenetic tree obtained by Bayesian Inference based on DNA sequences of the mitochondrial cytochrome *b* (1023 bp) grouped them within the Northern clade of the species but failed to separate them from the subspecies *V. b. sachalinensis*. The adder population from Southern Altay Mountains was grouped into the Northern clade together with the populations from Eastern Europe to Sakhalin Island of the Russian Far East and separated from the Balkan and the Italian clade. Bauwens and Claus (2019a, b) studied seasonal variation of mortality, detectability, body condition, intermittent reproduction, mortality patterns and lifetime breeding frequency of females in a population of the adder (*V. berus*). They used data collected during a capture–recapture study conducted over a 13-year period (2005–2017) in a large population of adders in northern Belgium and 18-year period (2000–2017) in a large population of European adders. Ursenbacher et al. (2009) found that *V. berus* exhibits a considerable genetic differentiation among populations even if these are not geographically isolated.

Efimov et al. (2008) determined the nucleotide sequences of mitochondrial genome fragments containing the genes coding for cytochrome oxidase subunit III and 12S ribosomal RNA of common European viper and Nikolsky's viper from various habitats. According to the sequencing data, the samples clustered into two groups. One group comprised *V. nikolskii* from Saratov oblast and the other, *V. berus* from the Chuvash Republic, Republic of Mordovia, and Samara and Penza oblasts (Efimov et al., 2008). *Vipera berus nikolskii* (Vedmederja et al., 1986) snakes are distinguished both by lepidotic and coloration traits. It is regarded as a *V. berus* subspecies in some publications (Zinenko et al., 2010, 2020). However, the analysis of the *V. nikolskii* venom revealed substantial differences from that of *V. berus* (Ramazanova et al., 2008; Kovalchuk et al., 2016). Thus, disintegrins, BPPs and CNPs were not found in *V. nikolskii* venom. Two heterodimeric PLA_2_s were isolated from the *V. nikolskii* venom (Ramazanova et al., 2008), but similar proteins are absent in *V. berus*. These findings support at the biochemical level that *V. nikolskii* may be an independent species. It was shown that *V. berus* hybridizes with *V. nikolskii* in Romania and Moldova (Zinenko et al., 2010) and with *V. aspis* in France (Guiller et al., 2017). The purpose of the review is to give a summary of different proteins and peptides isolated and characterized from the venoms of *V. b. berus* snakes. Some of these proteins and peptides are good models for drug design.

## 2 Toxicological characterization

The adder *V. berus* is the most widely distributed viper in Europe and it is known to cause more snakebite accidents than any other species of the genus Vipera (Reading, 1996; Chippaux, 2012; Di Nicola et al., 2021). Approximately 70% of the reported *V. berus* bites cause no or very mild effects in humans, and deaths rarely occur (Reading, 1996). The fatality by *V. berus* venom is rare throughout Europe (Warrell, 2005; Valenta et al., 2014; Paolino et al., 2020). Significant local tissue-damaging effects, including oedema, haemorrhage and myonecrosis have been observed after envenoming by *V. berus* snake (Calderón et al., 1993). Chippaux (2012) reported that the most common systemic symptoms of envenoming by *V. berus* are gastrointestinal upset with recurrent vomiting, circulatory instability, hypotension, and haematological disturbances. Valenta et al. (2014) analysed the most important epidemiological and clinical aspects of registered snakebites caused by a native common European viper *V. berus* in the Czech Republic over a period of 15 years (1999–2013). The envenoming was not usually associated with serious harm to the patients except for children. Hermansen et al. (2019) analysed envenomation of 219 patients by common European adder. The most patients bitten by *V. berus* had general symptoms such as oedema, erythema, nausea, vomiting and diarrhoea. A few case reports described more serious complications such as myocardial infarction, acute kidney failure, compartment syndrome. The lethal toxicity of *V. b. berus* venoms showed differences among individual snakes. Malina et al. (2017) revealed intra-population variability among venom samples from several individual European adders (*V. b. berus*) within a defined population in Eastern Hungary. *V. b. berus* population living in Eastern Hungary has predominantly neurotoxic neuromuscular activity (Malina et al., 2008, 2013, 2017). Zanetti et al. (2018) demonstrated that *V. berus* venom exerted no neurotoxic activity, differently from the venom of *V. aspis*. Their results are in line with the common belief that *V. berus* venom is devoid of neurotoxic activity, yet some *V. berus* subspecies (*V. berus bosniensis*) have been shown to cause neurological effects on animal models and humans (Westerström et al., 2010; Malina et al., 2011, 2017; Varga et al., 2018). Neurotoxicity is a most unusual and unexpected clinical feature of *V. berus* envenoming. Venom composition is highly variable among the different populations throughout the area of distribution of *V. berus.* Individual differences in venom pattern have been noticed, both gender-specific and age-related. Malina et al. (2008) hypothesized that these toxins are atavistic constituents of the berus-toxin, which disappeared from the venom of the other *V. b. berus* populations during venom evolution. Recently a systematic review involving variation in bite incidence and epidemiological characteristics of *V. b. berus* venom over a period of 49 years (1970–2019) has been published (Paolino et al., 2020).

The different parts of the Earth are contaminated with various radioactive isotopes due to the release of radioactive waste from nuclear fuel plants, accidents at atomic power stations and so on. Nedospasov and Cherkasov (1993) demonstrated that *V. b. berus* venom collected from contaminated districts of the former Soviet Union may be radioactive. It is highly likely that the snake venom is contaminated with radioactivity due to the environmental contamination.

## 3 Antivenoms

According to the World Health Organization recommendation, the most effective treatment for envenomation is the use of an antivenom serum. The specific antivenom consists of polyclonal antibodies isolated from hyperimmune animal serum/plasma. The mainstay of treatment for viper envenoming is the administration of polyclonal antibodies, known as monovalent or polyvalent antivenoms. Snakebite is usually treated by administering polyvalent antivenom derived from host sources such as horse, sheep, and donkey. The main advantage of using sheep lies in the excellence of their humoral immune response (Landon and Smith, 2003). The affinity purified ovine antivenom for the treatment of *V. berus* envenoming has been developed (Smith et al., 1992). The resultant product was three times more effective than the non-purified Fab in protecting mice against the lethal venom effects. An ovine affinity purified Fab antivenom was used in a clinical trial in Sweden to treat European adder (*V. b. berus*) envenoming (Sjostrom et al., 1996). Immunoassays were developed to measure *V. b. berus* venom and antivenom concentrations in clinical samples. A radioimmunoassay was developed, optimized, and validated to measure plasma levels of *V. b. berus* venom (Sjostrom et al., 1996). A rabbit antiserum to *V. berus* venom recognized all main venom bands by immunoblotting. This antiserum cross-reacted to a variable extent with several crotaline venoms, as assessed by enzyme immunoassay (Calderón et al., 1993).

Several Vipera spp antivenoms are produced in Europe, but there is little comparative information available for the antivenoms and none of them is licensed by the European Medicines Agency. Lamb et al. (2017) collected descriptive data on European viper antivenoms and assessed their relative effectiveness. The antivenom ViperaTAb® (MicroPharm Ltd, Newcastle Emlyn, United Kingdom) is a monovalent Fab antivenom derived from sheep hyperimmunized against *V. b. berus* venom. In addition to *V.b. berus*, ViperaTAb® shows clinical promise for treating snakebite of other European vipers (Casewell et al., 2014). ViperaTAb® antivenom appears to be effective and safe and should be administered as soon as possible for patients meeting clinical criteria outlined in guidelines for antivenom administration for *V. berus* envenoming (Lamb et al., 2021). After the cessation in manufacture of Zagreb antivenom, Public Health England recommends the use of ViperaTAb® for the management of *V. berus* envenomation. The safety and venom-neutralizing efficacy of Inoserp Europe - a new F(ab')₂ polyvalent antivenom, designed to treat envenoming by snakes in the Eurasian region - has been evaluated in mice and the results showed its appropriate neutralizing potency against the venoms of several species of Vipera, Montivipera, and Macrovipera. Inoserp Europe antivenom effectively neutralized five times the LD_50_ of all the venoms analysed, which demonstrates its cross-neutralization and paraspecific neutralization. The paraspecificity of the antivenom was demonstrated by its ability to neutralize venoms of species not included in the immunization schemes (Archundia et al., 2011; García-Arredondo et al., 2019). The Russian Microgen antivenom was able to neutralize lethal, haemorrhagic and PLA_2_ activities of *V. b. berus* venom. Al-Shekhadat et al. (2019) showed that the horse immunization procedure used to generate the Microgen antivenom was less effective than that applied for immunizing sheep for MicroPharm antivenom. The third generation antivenomics was applied to quantify the toxin-recognition landscape and the maximal binding capacity of the antivenom for each component of the venom. The antivenomics analysis revealed that 6.24% of the anti-*V. b. berus* F(ab')_2_ molecules fraction are toxin-binding antibodies, 60% of which represent clinically relevant antivenom molecules (Al-Shekhadat et al., 2019). Pla et al. (2020) showed that the monospecific anti-*V. b. berus* antivenom from Microgen® (Russia) displays remarkable paraspecificity towards the toxins of Dagestan blunt-nosed viper *Macrovipera lebetina obtusa* venom. The potency of the anti-*V. b. berus* antivenom could be improved 11.5 to 23 times by affinity chromatographic purification of its F(ab')_2_ antibodies. Recently Kurtovič et al. (2021) performed comparative analysis of the safety-related properties and efficacy of a panel of antivenoms against *V. ammodytes* and/or *V. berus* envenoming that are currently available, or in development for the European market. They revealed that Viperfav (Sanofi Pasteur SA, Lyon, France) had the best protection against lethal toxicity induced by the venoms of both *V. ammodytes* and *V. berus*. Viperfav is a polyspecific preparation based on F(ab')_2_ fragments against *V. aspis, V. berus,* and *V. ammodytes* venoms. In comparison to Zagreb antivenom Viperfav showed 4- to 5-fold weaker protective efficacy. Renewal of Zagreb antivenom production would be of great value (Kurtovič et al. 2021).

## 4 Proteomic studies

Venom toxins are classified into distinct protein families with diverse modes-of-action, which makes them a rich source for drugs targeting human proteins (Fry et al., 2009; Clark et al., 2019). Prior proteomic studies have demonstrated that *V. b. berus* venom contains serine proteinases (Siigur et al., 1979; Samel et al., 1987; Yukelson et al., 1995), metalloproteinases (Siigur et al., 1979, 2002; Samel and Siigur, 1990, 1995; Yukelson et al., 1995; Samel et al., 2003), PLA_2_ (Delori 1971, 1973; Boffa and Boffa 1976; Boffa et al., 1976; Siigur et al., 1979; Križaj et al., 1993; Yukelson et al., 1995), L-amino acid oxidases (Siigur et al., 1979; Samel et al., 2006), phosphodiesterases, phosphomonoesterases, 5′-nucleotidases, hyaluronidases (Siigur et al., 1979), NGF (Siigur et al., 1983, 1986, 1987), trypsin and chymotrypsin inhibitors (Siĭgur et al.,1988), disintegrins (Calvete et al., 2003), cysteine-rich secretory protein (Ramazanova et al., 2009) (see Table 1). Advances in DNA sequencing and proteomics have facilitated quantitative comparisons of snake venom composition. To make the current envenomation therapy more effective the proteomes of *V. b. berus* venoms have been analysed (Bocian et al., 2016; Latinović et al., 2016; Al-Shekhadat et al., 2019). The characterisation of the *V. b. berus* venom proteome produced rather variable results. The discrepancies (see Table 2) might be due to the geographical variations in the source of venoms or in the application of different protein identification techniques (Lomonte and Calvete, 2017; Malina et al., 2017; Varga et al., 2018; Di Nicola et al., 2021; Damm et al., 2021). Latinović et al. (2016) identified 10 different protein families in *V. b. berus* venom of Russian origin: serine proteases, metalloproteinases, natriuretic peptides, phospholipases A_2_, aspartic proteases, cysteine-rich secretory proteins, C-type lectins (snaclecs), L-amino-acid oxidases, disintegrins, and Kunitz-type protease inhibitors. Al-Shekhadat et al. (2019) investigated the toxic and enzymatic activities and determined 15 protein/peptide families in the *V. b. berus* venom. Phospholipases A_2_, serine proteinases, metalloproteinases, bradykinin-potentiating peptides, C-type natriuretic peptides, cysteine-rich secretory proteins, L-amino acid oxidase, C-type lectin-like (snaclecs), vascular endothelial growth factor, dimeric disintegrin, nerve growth factor, Kunitz-type protease inhibitors, 5′-nucleotidase, phosphodiesterase and hyaluronidase represent the components found in *V. b. berus* (Russia) venom (see Table 2). Bocian et al. (2016) analysed the venom of *V. b. berus* specimens collected from the Slovakian Republic. The most abundant proteins were phospholipases A_2_ (59% of all identified venom proteins) and serine proteinases (15%). Other venom components were snaclecs, CRISPs, LAAOs, SVMP, angiotensin-like peptides and 4 bradykinin-potentiating peptides. The composition of the Slovak *V. b. berus* venom is like the *V. nikolskii* venom with a very high content of PLA_2_ and a notably low abundance of SVMP. The proteins from *V. nikolskii* venom were categorized into 14 venom protein families (Kovalchuk et al., 2016). The most numerous classes were PLA_2_, SVMP, SVSP and VEGF, while disintegrins, BPPs and CNPs were absent in *V. nikolskii* venom (Table 2). Proteomic studies demonstrated the quantitative differences in the composition of *V. b. berus* venom from different geographical regions. Damm et al. (2021) reviewed all compositional venom studies (89 venom proteomes) of the so-called Old-World Vipers including *V. b. berus* venom.

**Table 1:** General characterization of components from *V. berus berus* venom.

Component name	Mass (kDa)	pI	Function/substrates	References
Enzymes				
Phosphodiesterase	100	4.0; 6.3	Hydrolysis of nucleic acids and nucleotides, depletion of cyclic, di- and trinucleotides	Siigur et al. (1979)
5′-nucleotidase	100	5.6	Hydrolysis of 5′-nucleotides, nucleoside liberation	Siigur et al., 1979; Aird, 2002
Phosphomonoesterase	150	nd	Hydrolysis of phosphomonoester bonds	Siigur et al., 1979; Rael, 1998
Hyaluronidase	73	nd	Hydrolysis of interstitial hyaluronan, diffusion of venom components	Siigur et al., 1979; Kudo and Tu (2001)
L-amino acid oxidase (homodimer)	57.7 126	4.8	Oxidative deamination of L-amino acids, induction of apoptosis, cell damage	Siigur et al., (1979), Samel et al. (2006)
Metalloproteinases				
Haemorrhagic metallo-proteinase (HMP)	56.3	6.3	Hydrolysis of proteins, haemorrhage, myonecrosis prey pre-digestion	Samel and Siigur (1990)
VBFXAE	38	3.5–4.5	Factor X activator, oxidized insulin B chain	Samel and Siigur (1995)
VBFXAEI	95.5	nd	Factor X activator, oxidized insulin B chain	Siigur et al. (2002)
VBFXAEII	126	nd	Factor X activator, oxidized insulin B chain, asocasein, gelatin, fibrinogen	Samel et al. (2003)
Serine proteinases				
Arginine esterase E1	38.5	4.0–4.6	Hydrolysis of BAEE, TAME, Pro-Phe-Arg-MCA	Samel et al. (1987)
Arginine esterase EII	41.0	3.3–3.9	Hydrolysis of BAEE, TAME, Pro-Phe-Arg-MCA, kinin-releasing activity	Samel et al. (1987)
Phospholipase A_2_ (France)	13.4	9.2	Hydrolysis of 2-acyl groups in 3-sn- phospho-glycerides, lipid membrane damage	Boffa et al. (1976)
Phospholipase A_2_ (Russia)	13.824	9.3	Hydrolysis of 2-acyl groups in 3-sn- phospho-glycerides, lipid membrane damage	Križaj et al., 1993
Phospholipase A_2_ (Hungary)	13.548–14.340^a^	nd	Hydrolysis of 2-acyl groups in 3-sn- phospho-glycerides, lipid membrane damage	Malina et al. (2017)
Phospholipase A_2__(Austria)_	13.550–14.2832^a^	nd	Hydrolysis of 2-acyl groups in 3-sn- phospho- glycerides, lipid membrane damage	Malina et al. (2017)
Phospholipase A_2_ (France)	13.824^b^	nd	Hydrolysis of 2-acyl groups in 3-sn- phospho-glycerides, lipid membrane damage, anticoagulant	Guillemin et al. (2003)
Nonenzymatic proteins/peptides				
CRISP	24.555	nd	Possibly blocks cNTP gated channels, Induces hypothermia, prey immobilization	Ramazanova et al. (2009); Yamazaki and Morita, 2004
Nerve growth factor	35	9.1–9.7	Promotes nerve fiber growth, differentiation of pheochromocytoma PC-12 cells	Siigur et al. (1986)
Dimeric disintegrin VB7	13.969	nd	Inhibits binding integrins to receptors, blocks the function of integrin α_5_β_1_	Calvete et al. (2003)
Trypsin inhibitor	7.3	>10	Inhibits trypsin K_i_ = 6.7 × 10^−11^	Siĭgur et al., 1988
Chymotrypsin inhibitor	7.3	9.9	Inhibits chymotrypsin K_i_ = 4.6 × 10^−10^	Siĭgur et al., 1988

nd – not detected.

a - 4–7 isoforms detected by MALDI-TOF MS in Hungarian *V. b. berus* venom; Austrian venom showed 5 isoforms with molecular masses between 13.550 and 14.2832.

b - The molecular mass of anticoagulant PLA_2_ has been detected by amino acid sequence of the *V. b. berus* protein that was identical with that of the PLA_2_ puriﬁed from *V. b. berus* venom (Križaj et al., 1993).

**Table 2:** Composition of protein and peptide families in the venom of *Vipera berus berus (Vbb*R*)* from Russia (Latinović et al., 2016), *Vbb*R1from Russia (Al-Shekhadat et al., 2019), *Vbb*S from Slovak Republic (Bocian et al., 2016), *Vipera nikolskii (Vnik)* (Kovalchuk et al., 2016).

Protein family	% of total venom proteins			
	*Vbb*R	*Vbb*R1	*Vbb*S	*Vnik*
Angiotensin-like	–	–	2	–
BNP	–	–	–	0.01
CNP	11	7.8	–	–
BPP	–	9.5	–	–
BP	–	–	–	0.15
Disintegrins	1	1.6	–	–
CTL/snaclec	2	3.5	6	4
SVMP inhibitor	4	–	–	–
KUN	0.07	2.6	–	0.66
CRISP	8	3.5	6	0.66
NGF	–	0.2	–	0.33
VEGF	–	4.3	–	8
AspP	0.12	–	–	–
PLA_2_	10	25.3**	59*	65
PLB	–	–	–	0.12
LAAO	2	7.3	9	0.08
SVSP	31	16.2	15	19
SVMP	19	17.2***	3.15	0.66
PDE	–	0.3-	–	-
5′-NT	–	0.3	–	0.88
Hyal	–	0.1	–	–
QC	–	0.07	–	–
TBP	–	–	–	0.68
Undefined	12	–	–	0.17

VbbR -*V. b. berus* venom obtained from the Serpentarium of the Central Trade Base ‘Zoo-obyedinenie’ Khimky (Moscow region, Russia).

VbbR1-venom obtained from LLC Siberian Serpentarium (Novosibirsk 630007, Russia).

59*-including 11% acidic PLA_2_, 47% basic PLA_2_ and 1% neutral PLA_2._

25.3**- this is dominated by at least 18 D49-PLA_2_s and a single S49-PLA_2_, which comprise 20.6% and 4.7% of the venom proteome.

17.2***- including 0.9% P1-type SVMP and 16.3% PIII-type SVMP.

BNP, B-type natriuretic peptide; CNP, C-type natriuretic peptide; BPP, bradykinin potentiating peptides; BP, blood protein; CRISP, cysteine-rich secretory protein; CTL, C-type lectin-like protein (snaclecs); KUN, Kunitz-type proteinase inhibitor; NGF, nerve growth factor; VEGF, vascular endothelial growth factor; AspP, aspartic protease; Hyal, hyaluronidase; LAAO, L-amino acid oxidase; 5′NT, 5′- nucleotidase; PDE, phosphodiesterase; PLA_2_, phospholipase A_2_; PLB, phospholipase B; QC, glutaminyl-peptide cyclotransferase; SVMP, snake venom metalloprotease; SVSP, snake venom serine protease; TBP, toxin biosynthesis proteins (including aminopeptidases).

## 5 Enzymatic proteins from *Vipera berus berus* venom

The major enzymatic proteins detected in *V. b. berus* venom include phospholipases A_2_, snake venom serine proteases, metalloproteinases, L-amino acid oxidases, phosphodiesterase, phosphomonoesterase, 5′-nucleotidase, hyaluronidases, ribonucleases (Siigur et al., 1979; Bocian et al., 2016; Latinović et al., 2016; Al-Shekhadat et al., 2019). Up to now phosphodiesterases, 5′-nucleotidases, ribonucleases and hyaluronidases are not isolated from *V. b. berus* venom. Enzymes detected by proteomics/venomics methods are given in Table 2.

### 5.1 Phospholipases A_2_ (PLA_2_s; EC 3.1.1.4)

Phospholipases A_2_ specifically catalyse the hydrolysis of the ester bond at the sn-2 position in glycerophospholipids, liberating free fatty acids and lysophospholipids. Phospholipases A_2_ are the most abundant proteins found in Viperidae snake venoms. Despite similarities in their structures and common catalytic properties, they exhibit a wide spectrum of pharmacological and toxicological functions, e. g. neurotoxicity, myotoxicity, cardiotoxicity, anticoagulant, and hemolytic activities. These proteins can display toxic eﬀects by diﬀerent mechanisms (Kini, 2003; Doley et al., 2010, reviews). The first toxic PLA_2_ from *V. berus* venom was purified by Delori (1971, 1973). An anticoagulant factor with phospholipase A_2_ activity has been isolated from *V. berus* venom (Boffa et al., 1976; Boffa and Boffa, 1976). It was a single-chain protein, formed by 119 amino acid residues, with a molecular weight of 13 400 and an isoelectric point of 9.2. Phospholipases A_2_ with molecular masses between 13 and 15 kDa from Eastern Hungary and Austria have been detected by MALDI-TOF MS. The Hungarian *V. b. berus* venom contained 4 to 7 isoforms with PLA_2_ activity, the Austrian adder venom showed 5 isoforms (see Table 1) (Malina et al., 2017). Three PLA_2_ isoforms have been detected by preparative isoelectric focusing of the Russian *V. b. berus* venom (Siigur et al., 1979). The basic, toxic phospholipase A_2_ was isolated from this venom and its primary structure was established. The enzyme is a single-chain protein with 14 Cys in positions characteristic for the phospholipase A_2_ subgroup IIA (Križaj et al., 1993). PCR-based method has been used to determine the genomic DNA sequences encoding phospholipases A_2_ from *Vipera aspis, Vipera aspis zinnikeri, V. b. berus* and *V. a. aspis* snake venoms (Guillemin et al., 2003). Jan et al. (2007) cloned and sequenced PLA_2_ transcripts from the venom glands of 21 European vipers from different species and subspecies including *V. b. berus*. The deduced amino acid sequence of the PLA_2_ from *V. b. berus* venom (France) was identical to that sequenced by Križaj et al. (1993). Samel et al. (2013) studied the inhibitory effects of PLA_2_ on human platelets, four different bacterial strains (gram-negative *Escherichia coli* and *Vibrio fischeri;* gram-positive *Staphylococcus aureus* and *Bacillus subtilis*) and on five types of cancer cells (PC-3, LNCaP, MCF-7, K-562 and B16–F10) *in vitro*. *V. b. berus* PLA_2_ inhibited collagen-induced platelet aggregation and the growth of gram-positive *Bacillus subtilis* whereas no growth inhibition was observed towards gram-negative *Escherichia coli*. The inhibitory action of PLA_2_ towards cancer cells depended on cell type. The enzyme inhibited the viability of K-562 cells and the cell death appeared apoptotic while it exhibited no inhibitory effect on LNCaP cells and only some effect (8%–20%) towards other studied cells (Samel et al., 2013). Recently, Siniavin et al. (2021) demonstrated that snake venom PLA_2_s exhibit strong antiviral activity against SARS-CoV-2 at nanomolar concentrations inhibiting the viral spike glycoprotein interaction with ACE2 of Vero E6 cells. *V. nikolskii* venom dimeric PLA_2_ and its subunits manifested especially potent virucidal effects, which were related to their phospholipolytic activity, and inhibited cell–cell fusion mediated by the SARS-CoV-2 spike glycoprotein. Snake PLA_2_s are promising for the development of antiviral drugs that target the viral envelope and could also prove to be useful tools to study the interaction of viruses with host cells (Siniavin et al., 2021).

### 5.2 Proteolytic enzymes

Comparative analysis of proteolytic activity in common viper *V. berus* venom samples received from different populations in European part of Russia and Ukraine showed about ten times differences in the highest and lowest activities (Malenyov et al., 2006). The venom of *V. b. berus* contains metallo- and serine proteinases that catalyse the digestion of tissue proteins and peptides (Siigur et al., 1979; Samel et al., 1987; Samel and Siigur, 1990, 1995; Yukelson et al., 1995).

#### 5.2.1 Snake venom metalloproteinases (SVMP)

SVMPs play key roles in the development of such symptoms as haemorrhage, oedema, hypotension, hypovolemia, inflammation, and necrosis (Fox and Serrano, 2005). SVMPs are Zn-dependent enzymes widely distributed in Viperidae venoms. They are synthesized as multidomain precursors and stored in the venom gland as inactive zymogens. SVMPs have been classiﬁed into three classes (PI, PII, and PIII) according to their multi-domain structure (Fox and Serrano, 2008; Markland and Swenson, 2013, reviews).

##### 5.2.1.1 Haemorrhagic metalloproteinase (HMP)

The occurrence of haemorrhage is one of the most striking consequences of envenomation by viperid venoms. The metalloproteinase isolated from *V. b. berus* venom demonstrated haemorrhagic activity with a minimum haemorrhagic dose about 4 μg per mouse. The caseinolytic activity of HMP was inhibited by EDTA, but not by PMSF. HMP is a glycoprotein with mol. mass of 56.3 kDa. Enzyme contains one zinc atom per molecule of protein. HMP hydrolyses casein, fibrinogen and splits the insulin B chain at the positions Ala14-Leu15, Tyr16-Leu17, His10-Leu11. In oxidized insulin B chain HMP digests the same bonds as HR-l proteinase from *Agkistrodon blomhoffi* venom (Nikai et al., 1986). It digests completely the A alpha chain and slowly the B beta chain of fibrinogen, the gamma chain is not digested (Samel and Siigur, 1990).

##### 5.2.1.2 Factor X activating enzymes

Human coagulation factor X is a serine protease zymogen, which circulates in blood as a two-chain molecule. A variety of factor X activators have been detected in snake venoms. Viperidae and Crotalidae venom activators are mainly metalloproteinases (Siigur and Siigur, 2006, 2010, reviews). Three factor X activating enzymes have been isolated from *V. b. berus* venom: VBFXAE (Samel and Siigur, 1995), VBFXAEI (Siigur et al., 2002) and VBFXAEII (Samel et al., 2003). VBFXAE is a single-chain glycoprotein with isoelectric points in the pH range of 3.5–4.5 containing 2 Ca^2+^ions per mole. The activator is inactive on synthetic substrates, on casein, prothrombin, and fibrinogen. VBFXAEI and VBFXAEII enzymes are high molecular mass proteinases (see Table 1). All three enzymes release factor Xa from human and bovine factor X, although the speciﬁc activities of *V. b. berus* venom enzymes are lower than these of *V. russelli* factor X activator (Takeya et al., 1992) and *V. lebetina* FXA (Siigur et al., 2001). All VBFXAEs cleaved factor X fragment NNLTRIVGG at positions Arg5–Ile6 and Leu3–Thr4. The activators also hydrolysed the insulin B-chain at the positions Ala14-Leu15 and Tyr16-Leu17. The speciﬁcity studies of factor X activating enzymes from *V. b. berus* venom demonstrate that these enzymes are nonspeciﬁc.

#### 5.2.2 Serine proteinases (SVSP)

Snake venom serine proteinases comprise a group of extensively studied toxins, widely found in the venom of snakes from Viperidae, Elapidae, and Crotalidae families. They are complex and multifunctional enzymes, acting primarily on haemostasis (Serrano and Maroun, 2005). A few serine proteinases have been detected in *V. b. berus* venom. Two glycosylated arginine ester hydrolases, designated EI and EII have been isolated and characterised from *V. b. berus* venom (see Table 1). Both enzymes were active towards the arginine esters BAEE and TAME. EI and EII differ in their activity towards kininogen, EII having high kinin-releasing activity, while EI has only weak activity against kininogen. Arginine ester hydrolases showed similar actions on Pro-Phe-Arg-MCA (Samel et al., 1987). Nedospasov and Rodina (1992) investigated age changes of amidolytic activity of *V. berus* venom using mixtures of chromogenic peptide substrates. Quantities of the venom (total protein content) and its proteolytic activity from snakes of different ages were compared. The venom composition of newly born adders was considerably different from the venom composition of young (12-month) adders of the same population.

### 5.3 L-amino acid oxidase (LAAO; EC 1.4.3.2)

L-amino acid oxidase (L-amino acid: O_2_ oxidoreductase) is a flavoenzyme that catalyses the stereospecific oxidative deamination of an L-amino acid to produce α-ketoacid, hydrogen peroxide and ammonia:

RCH(NH_3_^+^) COO^−^ + O_2_ + H_2_O → RCOCOO^–^ + NH_4_^+^ + H_2_O_2_

LAAO is responsible for the yellowish colour of venoms. A yellow colour has been detected in the fraction of preparative isoelectric focusing (pI 4.8) of *V. b. berus* venom (Siigur et al., 1979) and an L-amino acid oxidase isoform has been isolated (Samel et al., 2006). The enzyme is a non-covalently bound glycosylated homodimer with a monomeric molecular mass of 57.7 kDa. The purified protein catalysed oxidative deamination of L-amino acids; the most specific substrate is L-Phe. The best substrates among the studied 20 amino acids were: L-Met, L-Leu, L-Phe, L-Ile, L-Arg and L-His. Five amino acids, L-Ser, L-Pro, Gly, L-Thr and L-Cys, were not oxidized. The LAAO inhibited ADP- induced aggregation of platelets dose-dependently. Like all LAAOs, the LAAO of *V. b. berus* induced apoptosis of tumour cells (HeLa and K562). The inhibition of apoptosis by catalase suggested the role of hydrogen peroxide in the process (Samel et al., 2006). Recent reviews about snake venom LAAO are published in Guo et al. (2012), Tan et al. (2018), Hiu and Yap (2020).

## 6 Non-enzymatic proteins from *V. berus* venom

The non-enzymatic protein families identified from *V. b. berus* venom include disintegrins, nerve growth factor, cysteine-rich secretory proteins, snaclecs/C-type lectins, vascular endothelial growth factor, SVMP inhibitor, Kunitz-type inhibitors of trypsin and alpha-chymotrypsin, B- and C-type natriuretic peptides, bradykinin potentiating peptides, angiotensin-like peptides (Table 2).

### 6.1 Disintegrins

Disintegrins comprise a family of low-molecular-weight nonenzymatic integrin antagonists that are broadly distributed in viperid (Crotalidae and Viperidae) venoms (Calvete, 2005, 2013; Calvete et al., 2003, 2005). Disintegrins have originally been characterized as potent inhibitors of platelet aggregation. They bind to integrins also on the surface of malignant cells and cancer-associated angiogenic endothelial cells (McLane et al., 1998, 2008). Disintegrins can be divided into four groups according to their length (40–100 residues) and the number of disulphide bonds (4–8) (Calvete et al., 2003, 2005). A heterodimeric disintegrin, VB7, is detected in *V. berus venom* (Calvete et al., 2003) and its presence has been confirmed in proteomic survey. The disintegrin VB7 from *V. berus* venom was isolated by reverse phase HPLC and sequenced. VB7 consists of a 64 amino acid residue subunit A linked to a 63 amino acid residue subunit B by interchain disulphide bonds. The VB7 displays the RGD motif in subunit A and KGD motif in subunit B. It inhibited the adhesion of K562 cells, expressing the integrin α_5_β_1_, to immobilized ﬁbronectin, a component of blood plasma and extracellular matrix (Calvete, 2013). Fibronectin plays a crucial role in wound healing and formation of a blood clot to stop bleeding and protect the underlying tissue, therefore, upon envenomation, VB7 supposedly hinders these processes.

### 6.2 Nerve growth factor (NGF)

Nerve growth factor is a protein, which stimulates the differentiation and maintenance of sympathetic and embryonic sensory neurons. This protein, discovered by Cohen and Levi-Montalcini (1956) (Nobel Prize, 1986), plays a major role in the growth of nerve tissue, yet why this molecule is present in snake venom, in the first place remains an open question. An *in vitro* bioassay (with 8-day chick embryonic ganglia) has been used for detection of NGF activity in *V. b. berus* venom (Siigur et al., 1983). The *V. b. berus* venom NGF consists of multiple molecular forms with pIs in the interval 9.1–9.7. Nerve growth factor caused differentiation of pheochromocytoma PC12 cells (Siigur et al., 1986). The monoclonal antibodies to *M. l. turanica* NGF cross-reacted with NGF in *V.b. berus* venom (Saarma et al., 1984). Monoclonal antibodies to *M. l. turanica* venom nerve growth factor have been isolated (Arumäe et al., 1987) and these antibodies linked to BrCN-activated agarose have been used for purification of NGF from ten snake venoms including *V. b. berus* (Siigur et al., 1987). Anti-*M. l. turanica* NGF antibodies have been used for cross-reaction studies with 21 snake venoms. All studied venoms (including *V. b. berus*) contained NGF and the molecular masses of the NGFs have been determined (Trummal et al., 2011).

### 6.3 Cysteine-rich venom protein (CRISP-Vs)

Cysteine-rich proteins found in animal venoms (CRISP-Vs) are members of a large family of cysteine-rich secretory proteins (CRISPs). CRISP-Vs consist of a single polypeptide chain with a molecular weight of 23–26 kDa comprising 16 cysteine residues forming 8 disulphide bridges (Yamazaki and Morita, 2004). The cDNAs encoding CRISP-Vs from *V. berus* and *V. nikolskii* venoms have been cloned and sequenced (Ramazanova et al., 2009). The deduced mature CRISP-Vs amino acid sequences consist of 220 amino acid residues. The only difference between these two proteins is the presence of Lys92 instead of Glu92 in *V. berus*. Phylogenetic tree constructed in the result of analysis performed on 30 mature CRISP-V sequences (Ramazanova et al., 2009) is very similar to that obtained by Fry et al. (2008). They are phylogenetically closest to CRISP-V from *Protobothrops jerdonii*. Snake venom CRISPs inhibit ion channels and the growth of new blood vessels (angiogenesis). They also increase vascular permeability and promote inflammatory responses (leukocyte and neutrophil infiltration) (Tadokoro et al., 2020, review).

### 6.4 Kunitz-type serine protease inhibitors

The Kunitz-type serine protease inhibitors have been identified in the venoms of Viperidae and Elapidae. These 60 amino-acid long peptides are characterized by 6 conserved cysteine residues engaged in three disulphide bonds (Morjen et al., 2013). Inhibitors (I and II) with molecular masses of about 7000 Da and isoelectric points of greater than 10 and 9.9, respectively, have been isolated from the venom of *V. b. berus*. The inhibitor I prefers alpha-chymotrypsin (Ki = 4.6 × 10^−10^ M) for the formation of an enzyme inhibitor complex at a molar ratio of 1:1. The inhibitor II prefers trypsin (Ki = 6.7 × 10^−11^ M), forms an EI-complex at a molar ratio of 1:2, but also inhibits alpha-chymotrypsin (Ki = 1.4 × 10^−9^ M) and hog pancreatic kallikrein (Ki = 1.6 × 10^−8^ M) (Siĭgur et al., 1988). An overview of the structure-functional properties, pathophysiological significance, and possible therapeutic applications of protease inhibitors from snake venom has been presented by Thakur and Mukherjee (2017).

### 6.5 Other nonenzymatic proteins and peptides

*V. b. berus* contains nonenzymatic proteins that are not yet isolated and characterized but are detected by proteomics/venomics analysis. These are: angiotensin-like peptide (Bocian et al., 2016), NP (Latinović et al., 2016; Al-Shekhadat et al., 2019), BPP, VEGF (Al-Shekhadat et al., 2019), snaclecs (Bocian et al., 2016; Latinović et al., 2016; Al-Shekhadat et al., 2019) (Table 2). Snake venoms contain two types of C-type lectins based on structural features and functional properties: C-type lectin-like proteins and sugar binding snake lectins. C-type lectin-like proteins are composed of homologous heterodimers forming monomers or oligomers (αβ)_x_. They display various biological activities and are known to affect platelet aggregation. Clemetson proposed to call these proteins snaclecs (**sna**ke venom **c**type **lec**tin**s**) (reviews: Clemetson et al., 2009; Clemetson, 2010, 2021). Bocian et al. (2016) identified eight snaclecs in the *V. b. berus* venom, constituting 5.5% of venom proteins and this was the first finding of these proteins in this species. Up to now the snaclecs are not isolated and characterized from the venom.

## 7 Concluding remarks

The envenoming with *V. b. berus* venom is not usually associated with serious harm to the patients except for children. Venom composition is highly variable among the different populations throughout the area of distribution of snakes. Proteomic and functional analyses of *V. b. berus* venom indicate the presence of proteins belonging to at least 15 protein/peptide families, with predominance of PLA_2_s, serine- and metalloproteinases. PLA_2_s, SVMPs, SVSPs, LAAO, disintegrins, NGF, CRISP, Kunitz-type proteinase inhibitors have been isolated and characterised from *V. b. berus* venom. The high complexity, considerable enzymatic, antibacterial, and cytotoxic activities of *V. berus* venom imply as a promising source for new antibacterial and cytostatic agents. The toxins present in *V. berus* venom have also potential to serve as a basis for the design of new molecules with potential biotechnological application.

## Credit author statement

Jüri Siigur: conceived the theme, wrote the first draft of the manuscript; made subsequent revisions, reviewed the final draft of the manuscript before submission. Ene Siigur: revised the paper, reviewed the final draft of the manuscript before submission.

## Funding

This review needed no special funding. Both authors are retired scientists.

## Ethical statement

On behalf of, and having obtained permission from both authors, I declare that:

(a) the material has not been published in whole or in part elsewhere
(b) the paper is not currently being considered for publication elsewhere
(c) both authors have been personally and actively involved in work leading to the review. The authors have read the manuscript and agree to its publication in Toxicon X.

## Declaration of competing interest

The authors declare that they have no known competing financial interests or personal relationships that could have appeared to influence the work reported in this paper.
